# A Phase I Trial to Evaluate the Multiple-Dose Safety and Antitumor Activity of Ursolic Acid Liposomes in Subjects with Advanced Solid Tumors

**DOI:** 10.1155/2015/809714

**Published:** 2015-03-19

**Authors:** Zhengzi Qian, Xianhuo Wang, Zheng Song, Huilai Zhang, Shiyong Zhou, Jing Zhao, Huaqing Wang

**Affiliations:** Department of Lymphoma, Sino-US Center for Lymphoma and Leukemia, Tianjin Medical University Cancer Institute and Hospital, National Clinical Research Center of Cancer, Key Laboratory of Cancer Prevention and Therapy, Tiyuanbei, Huanhuxi Road, Hexi District, Tianjin 300060, China

## Abstract

Ursolic acid liposome (UAL), a new antitumor drug, has potential therapeutic value. However, limited clinical data exists regarding multiple-dose safety, antitumor activity, and the recommended dose (RD) of UAL for subjects with advanced solid tumors. All subjects were intravenously administered UAL for 14 consecutive days of a 21-day treatment cycle. Twenty-one subjects were enrolled in 1 of 3 sequential cohorts (56, 74, and 98 mg/m^2^) to evaluate multiple-dose tolerability and efficacy. Eight additional subjects were treated with UAL (74 mg/m^2^) to evaluate multiple-dose pharmacokinetics. No ≥grade 3 adverse events (NCI-CTC) were observed. Sixty percent subjects achieved stable disease after 2 treatment cycles. Multiple-dose pharmacokinetic analysis suggested UAL does not accumulate in the body. This trial demonstrates that UAL was tolerable, had manageable toxicity, and could potentially improve patient remission rates. A large phase II study is recommended to confirm these results (i.e., RD of 98 mg/m^2^).

## 1. Introduction

**Figure 1:**
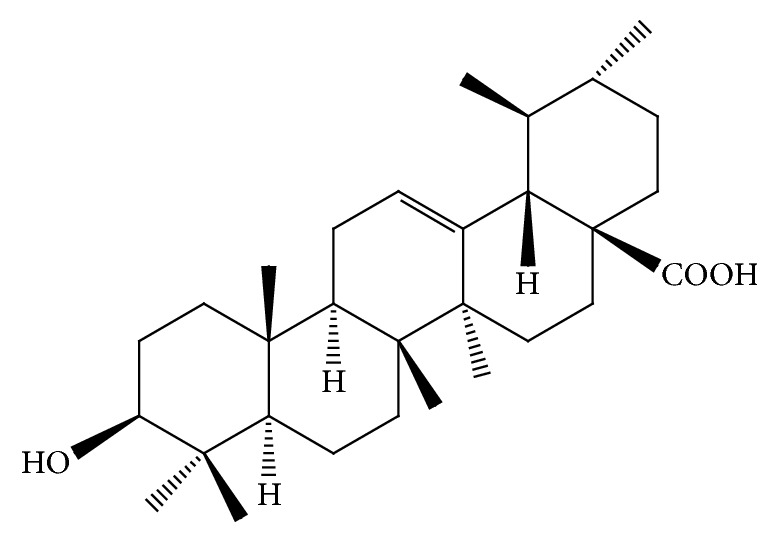
The chemical structure of ursolic acid.

Ursolic acid (UA) is a natural hydroxy pentacyclic triterpene compound (Figure 1) isolated from Chinese herbs including* Eriobotrya japonica*,* Rosmarinus officinalis*, and* Glechoma hederacea* [1, 2]. Previous studies have indicated that UA can induce apoptosis [3–5] and cell differentiation [6, 7], inhibit invasion and metastasis [8], and inhibit angiogenesis [9–11] in various tumors. UA treatment is also safe [12]. Thus, UA is a potentially valuable compound. However, the poor solubility of UA in hydrous solutions greatly limits its applications.

Liposomes have been utilized as a drug delivery system to overcome the poor solubility of UA, increase the therapeutic efficiency, reduce the side effects, and enhance the bioavailability of drugs that have been broadly applied [13–15]. Currently, ursolic acid liposomes (UALs) have been studied successfully and have been approved by the State Food and Drug Administration (SFDA) of China to enter clinical trials (number 2009L00634). We have previously published data regarding the maximum tolerated dose, dose-limiting toxicity (DLT), and pharmacokinetics of UAL in a single-dose administration study. The recommended doses in multiple-dose administration trials of UAL are 56, 74, and 98 mg/m^2^ [16]. In actuality, multiple-dose administration is usually adopted for most of drugs in clinic. Therefore, it is more important to study the effects of UALs in a multiple-dose administration trial.

The primary objective of this study was to evaluate the tolerability of UAL treatment and the recommended dose (RD) in a multiple-dose administration phase II trial consisting of subjects with advanced solid tumors. The second objective was to perform a preliminary assessment of the antitumor activity of UALs.

## 2. Materials and Methods

We performed a phase I, open-label, single center trial in subjects with advanced solid tumors. The SFDA of China and the Hospital Medical Ethics Committees approved the trial and it was conducted in accordance with the Declaration of Helsinki and the applicable local regulatory requirements and laws. A signed, written informed consent of the legal representatives and the consent of each patient were obtained before any study procedure was performed. UALs were supplied by Wuhan Li Yuanheng Medicine Technology Co. Ltd (Wuhan, China) as a freeze-dried powder for infusion. Each glass vial contained 3 mg of active drug. It was uniformly dispersed in 250 mL of 5% glucose solution before administration.

### 2.1. Patient Eligibility

Eligible subjects were aged 18–75 years with cytologically or histologically confirmed advanced solid tumors; they either refused standard therapies or standard effective therapies did not exist; and they had an Eastern Cooperative Oncology Group (ECOG) performance status (PS) of 0–2, a Karnofsky score ≥ 60%, and a life expectancy ≥ 3 months; practiced adequate contraception; had adequate hematological function (white blood cell (WBC) ≥ 4.0 × 10^9^/L, absolute neutrophil count (ANC) ≥ 2.0 × 10^9^/L, platelet count ≥ 100 × 10^9^/L, and hemoglobin ≥ 100 g/L); had adequate hepatic and renal function (alanine transaminase (ALT), aspartate transaminase (AST), and alkaline phosphatase (ALP) ≤ 2.5 the upper limit of normal (ULN) (or 5 × ULN for hepatic cancer/metastatic hepatic cancer), total bilirubin (TBIL) ≤ 1.5 × ULN, serum creatinine (CRE) levels of ≤ 1.5 × ULN, a creatinine clearance rate of ≤1.5 × ULN, and normal urea); and had normal pulmonary function.

### 2.2. Study Design and Treatment

Subjects were assigned to 1 of 3 sequential dose cohorts of UAL: 56, 74, or 98 mg/m^2^, administered via a 14-day consecutive intravenous 4 h infusion and given a rest for 7-day per 21-day cycle. Each cohort consisted of at least 3 subjects. Once all enrolled subjects had been monitored for 2 weeks and had no higher than grade 3 nonhematological toxicity or grade 4 hematological toxicity, the next dose was administered. The trial was terminated when ≥1/3 of the subjects experienced DLT, a severe adverse event (AE), or tumor progression. The DLT was defined as grade 4 thrombocytopenia, grade 4 neutropenia lasting for ≥7 days, febrile neutropenia, grade 4 anemia, or grade 3/4 nonhematological toxicity. Evaluated subjects were required to complete at least 1 cycle of treatment. After that, if subjects needed to continue treatment because they could not gain any benefit from other treatments, additional cycles were administered until disease progression or unacceptable toxicity occurred, or if the patient refused further treatment. Additional subjects were recruited in order to evaluate the pharmacokinetics of UAL treatment. These subjects were administered a dose of 74 mg/m^2^ of UAL via a consecutive, 14-day, intravenous 4 h infusion.

### 2.3. Tolerability and Toxicity

Tolerability and toxicity were evaluated in all subjects treated with at least 1 cycle of UAL therapy. Vital signs including body temperature, respiration, pulsation, and blood pressure were examined at screening and once a day thereafter. Hematological parameters (red blood cell, WBC, hemoglobin, ANC, and platelet), urine routines (urinary protein, glucose, erythrocyte, leukocyte, and urine bilirubin), and stool routines (fecal erythrocyte and fecal leukocyte) were tested, and an electrocardiogram was performed at screening and on the 14th day of the cycle. Blood biochemistries including ALT, AST, ALP, gamma-glutamyl transpeptidase (GGT), TBIL, direct-reacting bilirubin, total protein, GLU, lactate dehydrogenase, creatine kinase, bun urea nitrogen, CRE, UA, cholesterol, triglyceride (TG), high-density lipoprotein, low-density lipoprotein, K^+^, Na^+^, Ca^2+^, and Cl^−^ were examined at screening and then once a week thereafter. Fibrinogen (Fbg) and prothrombin time (PT) were examined at screening and during the 3rd week. To further evaluate the immune functions of subjects after UAL administration, we measured CD4/CD8 and natural killer (NK) cell activity in the circulation both at screening and on the 14th day. AEs were evaluated according to the National Cancer Institute Common Terminology Criteria for AEs (NCI-CTCAE) version 3.0.

### 2.4. Response Evaluation

Serial randomly subjects treated with at least 2 cycles were selected to evaluate the therapeutic efficacy of UALs. The tumor response was examined by using computerized tomography, magnetic resonance imaging, chest radiography, or ultrasonography according to the response evaluation criteria in solid tumors (RECIST) at the scheduled times (baseline and 2 cycles later) either until the tumor progressed or until the final visit. Complete response (CR), partial response (PR), stable disease (SD), and progressive disease (PD) were defined according to RECIST.

### 2.5. Multiple-Dose Pharmacokinetics

Blood samples for pharmacokinetic analysis were collected into heparinized tubes on the 1st and 14th days of the study, at various time points including 0, 0.5, 1, 2, and 4 h during infusion and 5, 15, and 30 min and 1, 1.5, 2, 3, 4, 6, 8, and 12 h after the end of infusion. Plasma was separated using centrifugation and then stored at –20°C until analysis.

UAL concentrations were measured using validated ultra-performance liquid chromatography/tandem mass spectroscopy (UPLC/MS/MS) methods as described previously [17]. In brief, chromatography was performed using a Waters Acquity UPLC BEH C_8_ column (100 × 2.1 mm, 1.7 *μ*m). The mobile phase consisted of acetonitrile and 10 mM ammonium formate (9 : 1, v/v) at a flow rate of 0.2 mL/min. The elution time was 3 m. Multiple-reaction monitoring was performed at *m*/*z*455.1 → 455.0 and *m*/*z*469.3 → 425.2 for UAL and glycyrrhetinic acid (internal standard), respectively, in negative ion mode with an electrospray ionization source. Estimates of pharmacokinetic parameters for UAL were derived from individual concentration-time data sets by noncompartmental analysis.

### 2.6. Statistical Considerations

Tolerability, toxicity, efficacy, and pharmacokinetic characteristics were explored and analyzed in detail. Noncompartmental pharmacokinetic parameters were determined from individual plasma concentration-time data using DAS version 2.1.1.

## 3. Results

### 3.1. Patient Characteristics

Twenty-one subjects (7 men and 14 women), aged 19–68 years (median age: 54 years), were enrolled in the study, and their characteristics at baseline are listed in Table 1. Twenty subjects (95%) had an ECOG performance status of 0-1. All subjects were treated with surgery (43%), radiotherapy (52%), chemotherapy (14%), and/or other therapies (67%). The study included 5 (24%) subjects with non-Hodgkin lymphoma, 5 (24%) subjects with Hodgkin lymphoma, 1 (5%) subject with renal carcinoma, 1 (5%) patient with hepatocellular carcinoma, 1 (5%) patient with gallbladder carcinoma, 2 (9%) subjects with breast cancer, 2 (9%) subjects with lung cancer, and 4 (19%) subjects with other cancers.

### 3.2. Tolerability and Toxicity

**Figure 2:**
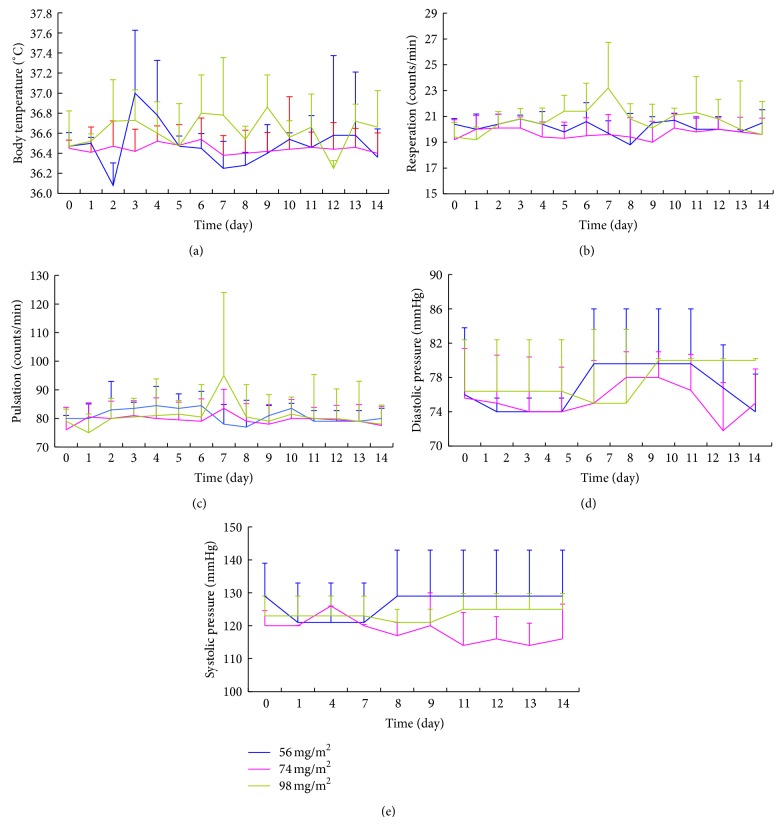
Vital sign data for the 3 cohorts at screening and throughout the infusion. (a) Body temperature, (b) respiration, (c) pulsation, (d) diastolic pressure, and (e) systolic pressure at the 3 different doses: 56 mg/m^2^ (*n* = 3), 74 mg/m^2^ (*n* = 14), and 98 mg/m^2^ (*n* = 4).

Tolerability and toxicity were evaluated for all subjects. The vital sign data showed that all values fluctuated within the normal range at every time point among the 3 cohorts (Figure 2). All hematological parameters (Fbg, PT) and results of electrocardiography and routine stool test were normal. Only 1 patient experienced grade 1 microscopic hematuria, while 2 subjects developed grade 1 proteinuria after 2 cycles of treatment with UAL (74 mg/m^2^).

Immune function tests showed no significant differences in CD4/CD8 at screening and on the 14th day (0.60 ± 0.31 and 0.82 ± 0.24, *P* > 0.05, 56 mg/m^2^; 0.82 ± 0.48 and 0.61 ± 0.24, *P* > 0.05, 74 mg/m^2^; 1.39 ± 0.96 and 1.23 ± 0.23, *P* > 0.05, 98 mg/m^2^). Significant differences in the NK cells were also not observed (18.40 ± 7.66 and 22.60 ± 5.97, *P* > 0.05, 56 mg/m^2^; 17.52 ± 11.57 and 20.87 ± 8.58, *P* > 0.05, 74 mg/m^2^; 17.91 ± 10.02 and 18.40 ± 7.50, *P* > 0.05, 98 mg/m^2^). These results suggested that the UAL did not affect patient immune function.

In addition, 3 (14%) subjects treated with 56 mg/m^2^ UAL developed a low-grade fever (grade 1) but then recovered after 2 h without any treatment (Table 2). Three (14%) subjects treated with 56, 74, and 98 mg/m^2^ UAL experienced grade 2 GGT elevation. Two (10%) subjects treated with 56 and 74 mg/m^2^ UAL experienced grade 1 abdominal distention. Finally, 1 (5%) patient had grade 2 ALT elevation. Other mild symptoms including AST and TG elevation, pruritus, arthralgia, and hypokalemia were also observed. However, no National Cancer Institute common toxicity criteria (NCI-CTC) ≥ grade 3 treatment-related AEs were observed. The most frequent AEs included pyrexia, GGT elevation, and abdominal distention. These results suggested that UAL was tolerable and safe among 3 dose cohorts after administration via a consecutive 14-day intravenous 4 h infusion every 21 days. Therefore, a UAL dose of 98 mg/m^2^ was considered the RD for a phase II trial.

### 3.3. Efficacy

As only 5 of 21 (23.8%) subjects preferred to receive and finish at least 2 cycles of UAL treatment, the evaluation of preliminary antitumor efficacy was limited. Three (60%) subjects achieved stable disease. One of these subjects had advanced renal carcinoma and had no significant change in the lesion after 2 cycles of treatment with 56 mg/m^2^ UAL. Another patient that had advanced hepatocellular carcinoma had no significant change in the lesion after 2 cycles of treatment with 74 mg/m^2^ UAL. Finally, the third patient had advanced lung cancer in which the lesion shrunk from 9.6 to 7.5 cm after 2 cycles of treatment with 98 mg/m^2^ UAL.

Two additional subjects, 1 with primary non-Hodgkin lymphoma and the other with breast cancer, showed PD after 2-cycle treatment with 74 mg/m^2^ UAL. No CR or PR was observed, which could be because the subjects had advanced stage tumors and did not benefit from other prior treatment schemes. Another possible explanation is that the number of subjects that could be evaluated was too small. Regardless, UAL does have the potential to improve the patient remission rate. A phase II study of a large number of subjects is recommended to confirm this finding.

### 3.4. Multiple-Dose Pharmacokinetics

Eight additional subjects were enrolled in the trial in order to investigate the pharmacokinetics of UAL therapy. The pharmacokinetic data (Table 3) following multiple-dose administration showed that the values of the elimination half-life (*t*_1/2_), maximum plasma concentration (*C*_max⁡_), area under the plasma concentration time curve (AUC_0→*t*_), and AUC_0→*∞*_ during the 1st day were 4.58 ± 2.04 h, 1589 ± 635 ng/mL, 5172 ± 1136 ng·h/mL, and 5498 ± 1525 ng·h/mL, respectively. They were 4.00 ± 1.27 h, 1211 ± 204 ng/mL, 4705 ± 873 ng·h/mL, and 4834 ± 933 ng·h/mL, respectively, during the 14th day. There were no significant differences in the values of *t*_1/2_, *C*_max⁡_, AUC_0→*t*_, and AUC_0→*∞*_ (*P* > 0.05) between days 1 and 14, suggesting that the pharmacokinetics were unaltered with multiple-daily dosing and that the UAL did not accumulate in the body. In addition, we found that there was a close relationship between the values of *C*_max⁡_ or AUC and AEs. The value of *C*_max⁡_ or AUC increased as the AEs (including hepatotoxicity and abdominal distension) increased in seriousness.

## 4. Discussion

This study demonstrated that UAL treatment of subjects with advanced solid tumors via multiple-dose and consecutive 14-day intravenous infusion every 21 days at doses of 56, 74, and 98 mg/m^2^ was safe. The results are consistent with preclinical information [12]. In addition, multiple-dose pharmacokinetics showed that the value of *C*_max⁡_ or AUC was associated with AEs. The value of *C*_max⁡_ or AUC was greater when the risk of AEs occurring in subjects was elevated. The reasons for this might be the following: when the value of *C*_max⁡_ is elevated, hepatocytes would be exposed to a high concentration of drug and would be stimulated to release serial enzymes including AST, ALT, and GGT. If the value of AUC was high simultaneously, the time of stimulation would be prolonged. Therefore, the risk of hepatotoxicity and gastrointestinal toxicity would become elevated. These results suggested that the manageable toxicity associated with UAL treatment could be further controlled via kinetic monitoring.

UA has been widely reported to have antitumor activities in preclinical studies [3–11, 18–21]. However, the clinical antitumor effects of UA or UAL have not been reported previously. In our study, the preliminary antitumor activity of UAL was evaluated for the first time in 5 subjects. Although no CR or PR occurred, SD was observed in 3 (60%) subjects with advanced solid tumors. Specifically, 1 lung cancer patient showed significant improvement and the lesion decreased in size (range, 9.6–7.5 cm) after 2 cycles of treatment with a UAL dose of 98 mg/m^2^. These results indicate UAL can potentially improve patient remission.

The pharmacokinetic data of UA in animals showed that *T*_1/2_ was about 4.3 h [22]. In this clinical trial, the mean *T*_1/2_ of UAL was 4.00–4.58 h, suggesting the *T*_1/2_ value was low so that it could rapidly be eliminated from blood. This phenomenon suggested that UAL did not accumulate in the body and that UAL must be infused repeatedly to keep the plasma-drug concentration steady and further enhance its antitumor effect.

## 5. Conclusions

In summary, the multiple-dose administration of UAL was tolerable with manageable toxicity. Further, the UAL did not accumulate in the body. We conclude that UAL has the potential to improve the patient remission rates. The recommended dose of UAL for a phase II clinical trial is 98 mg/m^2^.

## Figures and Tables

**Table 1:** Patient characteristics at baseline.

Characteristic	Subjects		
	56 mg/m^2^ (*n* = 3)	74 mg/m^2^ (*n* = 14)	98 mg/m^2^ (*n* = 4)
Gender, *n*			
Male	1	4	2
Female	2	10	2
Median age (range)	57 (49–59)	40.5 (19–68)	53.5 (42–59)
ECOG PS, *n*			
0	2	9	1
1	1	4	3
2	—	1	—
Type of tumor, *n*			
Non-Hodgkin lymphoma	1	3	1
Hodgkin lymphoma	—	5	—
Renal carcinoma	1	—	—
Hepatocellular carcinoma	—	1	—
Gallbladder carcinoma	1	—	—
Breast cancer	—	1	1
Lung cancer	—	—	2
Other	—	4	—
Prior therapy, *n*			
Surgery	0	7	2
Radiotherapy	3	7	1
Chemotherapy	0	2	1
Other therapy	0	11	3

ECOG: Eastern Cooperative Oncology Group; PS: performance status.

**Table 2:** Incidence of treatment-related adverse events.

AE, *N*	Number of subjects											
	56 mg/m^2^ (*n* = 3)			74 mg/m^2^ (*n* = 14)			98 mg/m^2^ (*n* = 4)			Total (*n* = 21)		
	G1	G2	≥G3	G1	G2	≥G3	G1	G2	≥G3	G1	G2	≥G3
Hepatotoxicity												
AST	—	—	—	1	—	—	—	—	—	1 (5%)	—	—
ALT	—	1	—	—	—	—	—	—	—	—	1 (5%)	—
GGT	—	1	—	—	1	—	—	1	—	—	3 (14%)	—
TG	—	—	—	1	—	—	—	—	—	1 (5%)	—	—
Abdominal distention	1	—	—	1	—	—	—	—	—	2 (10%)	—	—
Pruritus	—	—	—	1	—	—	—	—	—	1 (5%)	—	—
Arthralgia	—	—	—	1	—	—	—	—	—	1 (5%)	—	—
Low-grade fever	3	—	—	—	—	—	—	—	—	3 (14%)	—	—
Hypokalemia	—	—	—	1	—	—	—	—	—	1 (5%)	—	—

G1, G2, and G3 represent grade 1, grade 2, and grade 3, respectively, according to NCI-CTC grades.

AE: adverse event; —: no occurrence.

**Table 3:** Ursolic acid liposome pharmacokinetic parameters for the 1st and 14th days (mean ± standard deviation, SD; *n* = 8).

Parameter	Unit	Day 1	Day 14
		Mean ± SD	Mean ± SD
*t*_1/2_	h	4.58 ± 2.04	4.00 ± 1.27
*V*_*d*_	L/m^2^	88.60 ± 31.80	89.90 ± 28.10
CL	L/(h·m^2^)	14.40 ± 3.94	15.80 ± 3.05
AUC_(0–*t*)_	ng·h/mL	5172 ± 1136	4705 ± 873
AUC_(0–*∞*)_	ng·h/mL	5498 ± 1525	4834 ± 933
MRT_(0–*t*)_	h	3.34 ± 0.55	3.30 ± 0.31
MRT_(0–*∞*)_	h	4.31 ± 1.89	3.78 ± 0.70
*T*_max⁡_	h	3.00 ± 1.41	3.63 ± 1.06
*C*_max⁡_	ng/mL	1589 ± 635	1211 ± 204

## Abbreviations

UA: Ursolic acid
UAL: Ursolic acid liposome
RD: Recommended dose
SFDA: State Food and Drug Administration
MTD: Maximum tolerated dose
DLT: Dose-limiting toxicity
ECOG: Eastern Cooperative Oncology Group
PS: Performance status
AEs: AEs
NCI-CTCAE: National Cancer Institute Common Terminology Criteria for AEs
CT: Computerized tomography
MRI: Scan or magnetic resonance imaging
CR: Complete response
PR: Partial response
SD: Stable disease
PD: Progressive disease
UPLC/MS/MS: Ultra-performance liquid chromatography/tandem mass spectroscopy
EIS: Electrospray ionization source
Fbg: Fibrinogen
PT: Prothrombin time
*t*_1/2_: Elimination half-life
*C*_max⁡_: Maximum plasma concentration
AUC: Area under the plasma concentration time curve.
